# Cancer cells with trapped nuclei cut their way through the extracellular matrix

**DOI:** 10.1038/s41467-018-06351-6

**Published:** 2018-09-27

**Authors:** Emmanuel Dornier, Jim C. Norman

**Affiliations:** 1 CRUK Beatson Institute for Cancer Research, Garscube Estate, Glasgow, G61 1BD UK; 2 Institute of Cancer Sciences, University of Glasgow, Glasgow, G61 1QH UK

## Abstract

When an invading cancer cell attempts to pass through a hole in the extracellular matrix (ECM) which is too small for its nucleus, this generates physical tension. This tension is sensed by a nucleus–centrosome connection that activates trafficking of endosomal vesicles containing the matrix metalloprotease, MT1-MMP1 to the site of constraint. Recent evidence shows how focussed ECM degradation relieves the constraint and allows cancer cells to continue invading.

The nucleus is the densest of the cell’s organs and certainly one of the most rigid, thus posing a significant obstacle to movement through constricted spaces. Despite this, the nucleus is still deformable, and cells which traverse constrictions as part of their everyday function, such as the dendritic cells of the immune system, routinely deform their nuclei. Dendritic cells are highly motile, going back and forth from tissues and lymph nodes to harvest and present antigens to the immune system, and this circuitous journey entails negotiating constrictions imposed by lymphatic vessels and ECM which are substantially smaller than a cell nucleus. The nuclear lamina is a dense fibrillar protein network (composed largely of nuclear lamins) which associates with the inner face of the nuclear membrane, and confers rigidity to the nucleus^1^. Lamin levels dictate nuclear rigidity, and cells which habitually negotiate constrictions (such as dendritic cells and neutrophils) normally have low lamin levels and correspondingly flexible nuclei^2^. Therefore, when dendritic cells move through constricted spaces they can easily generate sufficient traction force to deform their nuclei. Additionally, cells can evolve mechanisms to actively disrupt their laminar network to further facilitate nuclear deformation. Indeed, there is a dense network of actin surrounding the nucleus linked to the cytosolic face of the nuclear envelope through a protein complex called the LINC complex. Through actin nucleation mediated by the Arp2/3 complex, dendritic cells can directly exert force on the lamina and thus alter the shape of their nucleus to facilitate movement through tissues and the lymphatic system without the need to resort to using proteases which would cause tissue damage^2^ (Fig. 1).

**Fig. 1:**
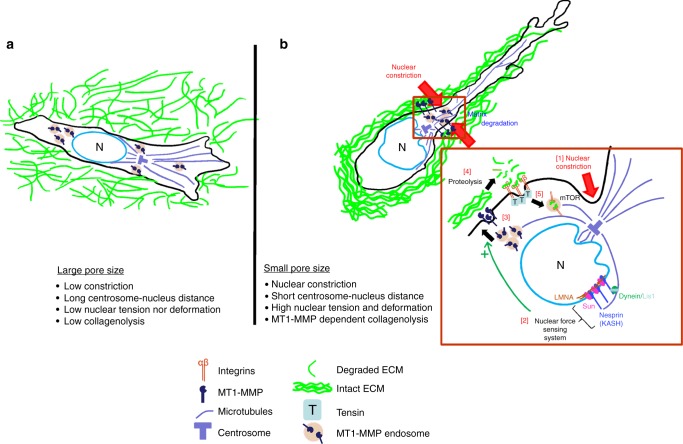
Nuclear constriction triggers delivery of vesicular MT1-MMP to the plasma membrane. **a** When cells invade through extracellular matrices (ECM) with large pores (pore size greater than the nucleus), there are few physical constraints on the nucleus and the centrosome–nucleus axis is not under tension resulting in a longer centrosome–nucleus distance. Low nuclear tension or little deformation are observed and the MT1-MMP delivery system is not triggered, as there is no need for collagenolysis to relieve physical constriction. **b** Cells invading into denser ECM, with pore size smaller than the nucleus, face a physical barrier to their movement. The matrix imposes a nuclear constriction [1] in the cell moving forward and creates nuclear tension that is sensed via the centrosome–nucleus linkage [2] which becomes stronger as evidenced by reduced centrosome–nucleus distance. This leads to the recruitment of intracellular MT1-MMP, stored in late endosomes, that then traffic to the site of physical constriction [3] on the plasma membrane. MT1-MMP then mediates ECM degradation to relieve the tension and make space for the nucleus to go through [4]. Integrins, also present in the same region of the plasma membrane, may then mediate uptake of the degraded matrix, in a tensin-dependent fashion. Degraded ECM then traffics to the late endosomes where it can support mTOR activation, a metabolic sensor [5]. Thus, focussed proteolysis at the points of nuclear constriction followed by ECM uptake through ligand-bound integrin internalisation may help cancer cells to scavenge nutrients to maintain energy balance

Recently, it has become clear that nuclear deformation threatens genome stability. Indeed, when dendritic cells deform their nuclei during migration this can lead to nuclear rupture^3,4^. This is evidenced by increased nucleocytoplasmic mixing, leading to DNA double-strand breaks. There are mechanisms in place to repair this deformation-induced damage to the nuclear envelope. Members of the ESCRT III family of endosomal regulators mediate resealing of the nuclear envelope during mitosis^5^. More recently it has been shown that ESCRT III also reseals holes made in the nuclear envelope that result from migration-induced nuclear deformation^3,4^. Thus, when ESCRT III levels are suppressed, dendritic cells are particularly susceptible to genomic instability (and the resulting cell death) during cell migration as they are unable to repair damage to their nuclei.

A key watershed during the progression of malignant disease is the acquisition of invasive characteristics which allow cancer cells to invade into neighbouring tissues, and to disseminate and colonise distant organs. During invasion, cancer cells need to squeeze through constrictions and this imposes stresses on the nucleus. Like dendritic cells, cancer cells can use actin-based mechanisms to disrupt the nuclear lamina^6^. Moreover, reduced levels of nuclear lamins (as is commonly observed in metastatic tumours) leads to increased nuclear flexibility^7^. Indeed, alterations in nuclear shape are a characteristic of malignant disease, and the degree of nuclear deformation may be used to rank tumours according to aggressiveness. Furthermore, cancer cells (like dendritic cells) can use ESCRT III to repair their nuclear envelopes and thus minimise genetic damage during invasion^3^.

Denais et al., reported that the damage to the nuclear envelope incurred during cancer cell migration is exacerbated by inhibition of ECM proteolysis^3^. This indicated that activation of matrix metalloproteases (MMPs) may provide one of the most straightforward routes to alleviating constrictions which lead to nuclear deformation, nuclear envelope rupture and DNA exposure to the cytoplasm. Previous work from the Weiss and Friedl laboratories, has shown that inhibition of MMP-mediated collagenolysis results in a higher proportion of cells deforming their nuclei to invade into constricted networks of fibrillar collagen^8^. In the recent publication in *Nature Communications*, Infante et al.^9^ have built upon this by demonstrating that cancer cells can respond to increased nuclear tension by mobilising MMPs from a vesicular pool. The authors have deployed an approach in which collagen plugs of defined pore size are generated by controlling the temperature at which the collagen is polymerised. Using this experimental setup, Infante et al. have shown that that MMP activity, and in particular MT1-MMP, is only required for cells to move through the ECM plugs with the tightest pores, thus confirming previous findings from the Weiss and Friedl labs. Moreover, they show that MT1-MMP-mediated proteolysis of the ECM appears to be associated with cells which are attempting to pass through an ECM constriction, but are frustrated in doing so because the pore is significantly smaller than the nuclear diameter. Interestingly, this proteolysis of the ECM does not occur randomly around this cell, but appears to be focussed to a region of the plasma membrane which is proximal to the ‘frustrated’ nucleus and which corresponds to the point where most physical tension would be expected to occur. Consistently, this focussed juxtanuclear proteolysis could be greatly reduced by knocking down lamins to render nuclei more deformable—a result which suggested a mechanistic connection between tension imposed on the nucleus and mobilisation of MT1-MMP.

MT1-MMP is a transmembrane protease which is localised in intracellular compartments corresponding, in the main, to late endosomes. There is now a substantial, and largely consistent, body of literature describing the machinery controlling the regulation of MT1-MMP trafficking^10^. MT1-MMP is continuously internalised from the plasma membrane and trafficked to late endosomes, and the rate at which it returns to the plasma membrane is controlled by Rab GTPases (most particularly Rab27), VAMP7, and CLIC3—all of which are key to MT1-MMP mediated invasiveness of cancer cells through dense 3D microenvironments^10–12^. Infante et al.^9^ visualised the distribution of MT1-MMP-containing vesicles late endosomes as cancer cells attempted to migrate through a small pore, and they found that this late endosomal population tended to congregate near the centrosome at the point of constriction. This led them to hypothesise that vesicular delivery of late endosomal MT1-MMP to the plasma membrane might be activated by, and targeted to, points of tension between the nucleus and the cytoskeleton and/or the centrosome.

The LINC complex connects the nucleus to the cytoskeleton and to the centrosome. The LINC complex is composed of SUN proteins present in the nuclear envelope which interact with Nesprins that connect to cytoplasmic elements, such as microtubules^1,7^. The LINC complex can also interact with cytoplasmic dynein and its regulator Lis1, to mediate movement of the nucleus towards the centrosome. The authors have shown that indeed Lis1 can be found at the nuclear envelope, and that this is dependent on the LINC complex. Disrupting the interaction of LINC with Dynein/Lis1 resulted in a decrease of invasion and collagenolysis. Under these conditions, polarisation of MT1-MMP endosomes was also lost, indicating that the LINC-Dynein complex can sense tension between the nucleus and the cytoskeleton to direct MT1-MMP endosomes to the site of nuclear constriction.

The perinuclear region is a very active zone in terms of vesicular trafficking. Indeed, it is now clear that the cytoskeletal protein tensin actively moves integrins engaged with ECM ligands to a point on the plasma membrane which is proximal to the nucleus, and it is here that extracellular matrix internalisation occurs^13^. This indicates the likelihood that following MT1-MMP-mediated proteolysis of ECM components at nuclear constrictions, tensin-dependent integrin internalisation may then subsequently act to internalise the proteolysed ECM. ECM internalisation via this pathway is promoted by withdrawal of nutrients, and enables cells to maintain active mTOR on their late endosomes when nutrients are scarce. Thus, focussed proteolysis at the points of nuclear constriction followed by ECM uptake through ligand-bound integrin internalisation may help cancer cells to scavenge nutrients to maintain energy balance in addition to alleviating nuclear deformation to protect the genome of cancer cells during invasion. This work highlights the possibility that targeting the machinery linking nuclear tension with the exocytic apparatus may be useful in our quest to expose metabolic vulnerabilities in cancer.
